# Synergistic effects of leucine and resveratrol on insulin sensitivity and fat metabolism in adipocytes and mice

**DOI:** 10.1186/1743-7075-9-77

**Published:** 2012-08-22

**Authors:** Antje Bruckbauer, Michael B Zemel, Teresa Thorpe, Murthy R Akula, Alan C Stuckey, Dustin Osborne, Emily B Martin, Stephen Kennel, Jonathan S Wall

**Affiliations:** 1 NuSirt Sciences Inc, 11020 Solway School Rd, Knoxville, TN, 37931, USA; 2 Department of Nutrition, University of Tennessee, 1215 W. Cumberland Ave, Knoxville, TN, 37996, USA; 3 Departments of Radiology, University of Tennessee Graduate School of Medicine, 1924 Alcoa Highway, Knoxville, TN, 37920-6999, USA; 4 Department of Medicine, University of Tennessee Graduate School of Medicine, 1924 Alcoa Highway, Knoxville, TN, 37920-6999, USA

**Keywords:** Diabetes, HMB, Inflammatory stress, Insulin-resistance, Leucine, Obesity, Resveratrol, Sirt1, Sirt3, Synergy

## Abstract

**Background:**

Sirtuins are important regulators of glucose and fat metabolism, and sirtuin activation has been proposed as a therapeutic target for insulin resistance and diabetes. We have shown leucine to increase mitochondrial biogenesis and fat oxidation via Sirt1 dependent pathways. Resveratrol is a widely recognized activator of Sirt; however, the biologically-effective high concentrations used in cell and animal studies are generally impractical or difficult to achieve in humans. Accordingly, we sought to determine whether leucine would exhibit synergy with low levels of resveratrol on sirtuin-dependent outcomes in adipocytes and in diet-induced obese (DIO) mice.

**Methods:**

3T3-L1 mouse adipocytes were treated with Leucine (0.5 mM), β-hydroxy-β-methyl butyrate (HMB) (5 μM) or Resveratrol (200 nM) alone or in combination. In addition, diet-induced obese mice were treated for 6-weeks with low (2 g/kg diet) or high (10 g/kg diet) dose HMB, Leucine (24 g/kg diet; 200% of normal level) or low (12.5 mg/kg diet) or high (225 mg/kg diet) dose resveratrol, alone or as combination with leucine-resveratrol or HMB-resveratrol.

**Results:**

Fatty acid oxidation, AMPK, Sirt1 and Sirt3 activity in 3T3-L1 adipocytes and in muscle cells, were significantly increased by the combinations compared to the individual treatments. Similarly, 6-week feeding of low-dose resveratrol combined with either leucine or its metabolite HMB to DIO mice increased adipose Sirt1 activity, muscle glucose and palmitate uptake (measured via PET/CT), insulin sensitivity (HOMA_IR_), improved inflammatory stress biomarkers (CRP, IL-6, MCP-1, adiponectin) and reduced adiposity comparable to the effects of high dose resveratrol, while low-dose resveratrol exerted no independent effect.

**Conclusion:**

These data demonstrate that either leucine or its metabolite HMB may be combined with a low concentration of resveratrol to exert synergistic effects on Sirt1-dependent outcomes; this may result in more practical dosing of resveratrol in the management of obesity, insulin-resistance and diabetes.

## Introduction

Leucine exerts a well established stimulatory effect on protein synthesis via both mTOR-dependent and independent pathways and an anti-proteolytic effect both in muscle and other tissues such as adipose tissue [1-3]. The energetic cost of protein synthesis and turnover may result in increased fatty acid oxidation and net energy utilization. Consistent with this concept, we have demonstrated leucine to promote energy partitioning from adipocytes to skeletal myotubes in co-culture systems, resulting in net reductions in adipocyte lipid storage and increases in muscle fatty acid oxidation [4,5]. Thus, leucine may attenuate adiposity and promote weight loss during energy restriction [6,7]. These effects are, in part, mediated by Sirt1-dependent pathways and associated with stimulation of mitochondrial biogenesis and increased oxygen consumption [5]. Although the underlying molecular mechanism is still unclear, our data demonstrate leucine and its metabolites, α-keto-isocaproic acid (KIC) and β-Hydroxy-β-Methylbutyrate (HMB), to activate Sirt1 directly as demonstrated in a cell-free system [8], as well as to activate Sirt1-dependent signaling pathways for fat oxidation and insulin signaling, and to attenuate pathways of oxidative and inflammatory stress [9]. Since these pathways are also modulated by other sirtuins such as the mitochondrially located Sirt3, it is possible that leucine and its metabolites may modulate Sirt3 as well.

The sirtuins Sirt1 (Silent Information Regulator Transcript 1) and Sirt3 belong to a class of NAD^+^-dependent protein deacetylases involved in the regulation of energy metabolism and cellular survival [10,11]. While Sirt3 is located in the mitochondria, Sirt1 is mainly found in the nucleus. Both sense energy status via the NAD^+^/NADH ratio and modify the acetylation level of histones and proteins such as p53, NF-κB and FOXO [12,13]. Sirt1 and Sirt3 stimulation leads to activation of mitochondrial biogenesis and metabolism, resulting in increased fatty acid oxidation and decreased reactive oxygen species (ROS) production [14]. Since mitochondrial dysfunction has been suggested to play a role in the development of metabolic diseases such as insulin resistance and diabetes, sirtuin activators may have therapeutic potential [15,16].

Resveratrol, a plant polyphenol found in the skin of red grapes and in other fruits, has been reported as a Sirt1 activator, mimicking the effects of caloric restriction on life span [17,18]. Recent evidence suggests that this Sirt1 activation may not be directly mediated, as suggested before, but rather indirectly by inhibiting cAMP phosphodiesterase resulting in upregulation of AMPK and increased levels of NAD^+^[19]. However, others have argued that this may be the case only at high concentrations (50 μM) while AMPK activation at low concentration seemed to be Sirt1-dependent [20]. Despite this uncertainty regarding mechanism of action, resveratrol consumption has been shown to exert beneficial metabolic effects on glucose homeostasis and to protect against metabolic diseases such as diabetes [21,22]. However, some of these effects are only achieved at dosages which are difficult to obtain by humans. While anti-inflammatory and anti-oxidant effects are found in the low micromolar range, other effects require higher concentrations (>50 μM to mM range) [23]. Because of its limited bioavailability and rapid metabolism, detectable (if at all) plasma concentrations in humans are usually much lower than the micromolar concentrations used in in vitro studies [24-26]. Therefore, results from cell/animal studies with such high concentrations of resveratrol are not readily translated to human outcomes.

Since leucine and resveratrol both converge on the Sirt1 pathway, either directly or indirectly, the premise of this work is that co-administration of leucine with resveratrol will result in synergistic activation of Sirt1 and possibly Sirt3, thereby lowering the levels of resveratrol required to exert significant metabolic benefit. Accordingly, we studied concentrations of resveratrol and leucine and it’s metabolites that exert little if any independent effects on sirtuin signaling and that are readily achievable following consumption of a high protein meal (0.5 mM leucine) and a single doses of 0.5 g resveratrol or repeated dose of 150 mg resveratrol (200 nM), respectively [27-29]. Similarly, we have chosen the low-dose concentration of resveratrol (12.5 mg/kg diet) in the animal study to be lower than that of other comparable low dose resveratrol mouse studies, which was calculated to be between 50 mg and 100 mg/kg diet [17,30]. Since some of the leucine effects are also mediated by its metabolite β-Hydroxy-β-Methylbutyrate (HMB), we included treatment groups with HMB and resveratrol in this study as well.

## Material and methods

### Experimental approach

Based on our previous data demonstrating significant effects of leucine and its metabolite HMB on Sirt1 activation and fat metabolism, the primary purpose of this study was to investigate whether leucine and/or HMB synergize with resveratrol, as another Sirt1 activator, in Sirt1 activation and downstream effects. In addition, we wanted to explore possible effects on other sirtuins such as Sirt3. This was first done in cell culture and then extended to an in vivo mouse study where we also measured downstream effects of Sirt1 activation on insulin sensitivity, and glucose and palmitate uptake. Since Sirt1 also modulates oxidative and inflammatory stress, we included plasma markers of both as well. In addition, we included metabolic chamber studies to measure overall heat production and oxygen consumption. Depending on the complexity of experiments, we could not include all possible treatment combinations in all of the experiments. For the same reason, we did not incorporate other branched-chain amino acids as controls for non-specific effects of leucine to our experiments, as we have previously demonstrated that these exert no independent effects in these systems [3,4,8].

### Cell culture

3T3-L1 pre-adipocytes were incubated at a density of 8000 cells/cm^2^ (10 cm^2^ dish) and grown in the absence of insulin in Dulbecco’s modified Eagle’s medium (DMEM,25 mM glucose) containing 10% fetal bovine serum (FBS) and antibiotics (1% penicillin-streptomycin)(adipocyte medium) at 37°C in 5% CO_2_ in air. Confluent pre-adipocytes were induced to differentiate with a standard differentiation medium consisting of DMEM-F10 (1:1, vol/vol) medium supplemented with 10% FBS, 250 nM dexamethasone (DEXA), isobutylmethylxanthine (IBMX) (0.5 mM) and antibiotics. Pre-adipocytes were maintained in this differentiation medium for 3 days and subsequently cultured in adipocyte medium for further 8 to 10 days to allow at least 90% of cells to reach fully differentiation before treatment. Media was changed every 2–3 days, differentiation was determined microscopically via inclusion of fat droplets.

C2C12 muscle cells were incubated at a density of 8000 cells/cm^2^ (10 cm^2^ dish) and grown in Dulbecco’s modified Eagle’s medium (DMEM) containing 10% fetal bovine serum (FBS) and antibiotics (adipocyte medium) at 37°C in 5% CO2 in air. Cells were grown to 100% confluence, changed into differentiation medium (DMEM with 2% horse serum and 1% penicillin– streptomycin), and fed with fresh differentiation medium every day until myotubes were fully formed (6 days).

Treatment concentrations for all experiments were 200 nM Resveratrol, 0.5 mM Leucine, 5 uM HMB in high (25 mM) glucose unless otherwise stated; incubation time was between 4 and 24 h, depending on experiment.

### Sirt1 activity

Sirt1 activity was measured by using the Sirt1 Fluorimetric Drug Discovery Kit (BML-AK555, ENZO Life Sciences International, Inc. PA, USA). The sensitivity and specifity of this assay kit was demonstrated by Nin et al. [31]. They showed no detectable activity in Sirt1 knockout embryonic fibroblasts, demonstrating that the enzymatic activity measured by this assay is not present in cellular extracts that lack Sirt1.

In this assay, Sirt1 activity is assessed by the degree of deacetylation of a standardized substrate containing an acetylated lysine side chain. The substrate utilized is a peptide containing amino acids 379–382 of human p53 (Arg-His-Lys-Lys[Ac]), an established target of Sirt1 activity; Sirt1 activity is directly proportional to the degree of deacetylation of Lys-382. Samples were incubated with peptide substrate (25 μM), and NAD^+^ (500 μM) in a phosphate-buffered saline solution at 37°C on a horizontal shaker for 45 minutes. The reaction was stopped with the addition of 2 mM nicotinamide and a developing solution that binds to the deacetylated lysine to form a fluorophore. Following 10 minutes incubation at 37°C, fluorescence was read in a plate-reading fluorimeter with excitation and emission wavelengths of 360 nm and 450 nm, respectively. Resveratrol (100 mM) served as a Sirt1 activator (positive control) and suramin sodium (25 mM) as a Sirt1 inhibitor (negative control). The endogenous Sirt1 activity in muscle cell and mouse white adipose tissue (WAT) was measured in a modified assay using 5 μl of cell or tissue lysate. The lysates were prepared by homogenizing cells or frozen tissue in ice-cold RIPA buffer plus protease inhibitor mix (MP Biomedicals LLC). After 10 min incubation on ice, the homogenate was centrifuged at 14,000 x *g* and the supernatant was used for further experiments. Data for endogenous Sirt1 activation were normalized to cellular protein concentration measured via BCA-assay.

### Sirt3 activity

For assaying Sirt3 activity, mitochondrial protein was isolated from treated adipocytes using the Mitochondria Isolation Kit from Sigma (Saint Louis, MO, USA) and Sirt3 activity was assessed by fluorometric measurement of deacetylation of a Sirt3 substrate (Sirt3 Fluorimetric Drug Discovery Kit, Enzo Life Sciences International, Inc. PA, USA), as described for Sirt1.

### AMPK activity

AMPK activity in 3T3-L1 adipocytes was measured via the AMPK Kinase Assay Kit (CycLex Co., Ltd., Nagano, Japan) according to manufacture’s instruction. This assay provides a non-isotopic, sensitive and specific method in form of an ELISA and uses anti-phospho-mouse IRS-1 S789 monoclonal antibody and peroxidase coupled anti-mouse IgG antibody as a reporter molecule. The amount of phosphorylated substrate is determined by measuring absorbance at 450 nm. Differentiated cells were incubated with indicated treatments for 24 h. Cells were washed three times with ice-cold PBS, then lysed in Cell Lysis Buffer for 90 minutes on ice, centrifuged at 3,500 rpm for 15 min at 4^0^C. Then 10 μl of clear supernatant was used for each assay experiment. Purified recombinant AMPK active enzyme was included as a positive control for phosphorylation. To calculate the relative AMPK activity of the samples, an inhibitor control with Compound C for each sample was included once and inhibitor control absorbance values were subtracted from test sample absorbance values.

### Fatty acid oxidation

Fatty acid oxidation was measured using ^3^H]-palmitate, as previously described [4]. Briefly, cells were rinsed twice with phosphate-buffered saline (PBS) and incubated in substrate mixture containing 22 uM unlabeled palmitate plus 5 uCi ^3^H]-palmitate in Hank’s basic salt solution containing 0.5 mg/ml BSA for 2 h. The reaction medium was then collected and treated with 0.2 ml 10% trichloracetic acid. The protein precipitate was removed by centrifugation while supernatants were treated with 6 N NaOH and then applied to a poly-prep chromatography column with 1 ml Dowex-1. The ^3^H_2_O passed through the column and the following 1 ml of water wash was collected and radioactivity was measured with a liquid scintillation counter. Protein content of the cell monolayer was measured using Bradford protein assay reagents and used for normalization.

### Animals and Diet

Six-week-old male c57/BL6 mice (Harlan Laboratories, Indianapolis, IN) were fed a high-fat diet with fat increased to 45% of energy (Research Diets D12451) for 6 weeks to induce obesity. At the end of this obesity induction period, animals were either maintained on the control diet or randomly divided into one of the diet treatment groups (10 animals per group) which were supplemented with resveratrol (low dose (12.5 mg/kg diet) or high dose (225 mg/kg diet), calcium salt of hydroxymethylbutyrate (Ca-HMB: low dose (2 g/kg diet) or high dose (10 g/kg diet)) or leucine (increased to 24 g/kg diet), alone or in combination. All diets were isocaloric (4.7 kcal/g).

The animals (two/cage) were housed in polypropylene cages at a room temperature of 22°C ± 2°C and regime of 12 h light/dark cycle. The animals had free access to water and their experimental food throughout the experiment. All animals were checked daily for any signs of disease or death and moribund animals (as defined by the facility veterinarian) were humanely euthanized. Overall, one animal died before start of intervention, and three moribund animals were euthanized (two before start of intervention, one from low Resv/low HMB group). Weight was measured to the nearest gram at the beginning of the experiment and then weekly until the end of the study. At the end of the treatment period (6 weeks) all animals were humanely euthanized with isoflurane overdose, followed by cervical dislocation to assure death. Blood was immediately collected by cardiac puncture. The excised tissues were immediately weighed and used for further studies. All sacrifices were done in the morning with 7 animals per day with a minimum of 4 to 5 hours fast.

The University of Tennessee Graduate School of Medicine is a AAALAC-I-accredited institution. This study and all animal procedures were performed under the auspices of an Institutional Animal Care and Use Committee-approved protocol and in accordance with PHS policy and recommendations of the Guide.

### Oxygen consumption/substrate utilization

At the end of the obesity induction period (day 0 of treatment) and at 2 and 6 weeks of treatment, oxygen consumption and substrate utilization was measured via metabolic chambers using the Comprehensive Lab Animal Monitoring Systems (CLAMS, Columbus Instruments, Columbus, OH) in subgroups of each treatment group. Each animal was placed in individual cages without bedding that allow automated, non-invasive data collection. Each cage is an indirect open circuit calorimeter that provides measurement of oxygen consumption (VO_2_), carbon dioxide production (VCO_2_), and concurrent measurement of food intake. All mice were acclimatized to the chambers for 24 h prior to the experiment and maintained under the regular 12:12 light:dark cycle with free access to water and food. All experiments were started in the morning and data were collected for 24 h. Each chamber was passed with 0.6 l of air/min and was sampled for 2 min at 32-minute intervals. Exhaust O_2_ and CO_2_ content from each chamber was compared with ambient O_2_ and CO_2_ content. Food consumption was measured by electronic scales. The respiratory exchange ratio (RER) was calculated from the ratio between carbon dioxide production and oxygen consumption (RER = VCO_2_/VO_2_) before weight normalization or mass correction. Heat production (kcal/h) was derived from a calculated calorific value based on the observed RER which was then multiplied by the observed VO_2_ (heat (kcal/h) = [3.815 + (1.232 x RER)] x VO_2_.

### microPET/CT - measurement of tissue glucose and palmitate uptake

After 6 weeks of treatment intervention, subgroups of each treatment diet group (5 animals/group, 35 animals total) were used to measure whole body glucose and palmitate uptake via PET/CT Imaging. To visualize these compounds using microPET imaging, the glucose or palmitate was labeled with fluorine-18 (^18^F, 110 min half life). Based on previous experience with palmitate imaging, after fasting overnight, each mouse was injected iv with an average of ~124 μCi of each tracer, then be left for a period of time ~ 1 h to allow the uptake of the tracer [32-34].

After that time, the animals were anesthetized using 1-3% isoflurane delivered by nose cone or in a mouse-sized induction chamber purpose-built for small animal imaging protocols and the PET/CT images were acquired using an Inveon trimodality, PET/SPECT/CT platform (Siemens Medical Solutions, Knoxville, TN). PET data were acquired using an energy window of 350 – 650 keV and histogrammed using a 3D rebinning algorithm using a span of 3 and ring difference of 79. Data were reconstructed using a 2D ordered subset expectation maximization (OSEM) algorithm. CT data were collected using X-ray tube settings of 80 kVp and 0.5 mA. Exposure times were adjusted based on manufacturer recommendations resulting in a 225 ms exposure time per projection. Each image comprised 360 projections acquired at 1° intervals and was reconstructed using a modified Feldkamp algorithm. During image acquisition, the mice were kept warm using a thermostatically controlled heated bed and were treated with ophthalmic ointment prior to scanning. Following the live scan the mice were returned to their cage and revived. Mice were monitored constantly during this time. Each animal received both probes 48 h apart in the same order (first palmitate, second glucose) to ensure complete decay of the previous probe. Following live data acquisition of the last probe the mice were sacrificed by isoflurane overdose and organs harvested for further experiments.

Quantitation of radiotracer uptake in defined regions of adiposity was achieved by determining the standard uptake value (SUV) using the PET image data. This quantitative value is the most commonly used representation of PET data and is often seen in conjunction with ^18^FDG studies and has been used here to quantify ^18^F-palmitate also [35].

PET image data were corrected for radionuclide decay to the acquisition start time. Images were further calibrated using a scaling factor determined, using standard procedures resulting in image data expressed in Bq/ml. Regions of interest were drawn on the subject images to include perirenal, omental or subcutaneous fat depots as well as regions of outer and inner leg muscle using IRW (Siemens Medical Solutions). SUVs were calculated using the following equation

(1) $$SUV=\frac{{\bar{Activity}}_{\mathrm{ROI}}}{InjectedDose}xSubjectWeight$$

where the injected dose was decay corrected from the time of injection to the image acquisition start time.

Mean and maximum SUVs were calculated for each ROI and the “muscle-to-adipose tissue” ratio for the each were calculated. Three mice deemed to be diabetic by fasting blood glucose were omitted from the analysis of FDG uptake. Additionally, a blinded review of the PET image data was performed (D.O. and E.M) to identify mice in which abnormal FDG distribution was observed and these mice were also removed from the analysis (n = 4).

### Calculation of visceral adipose volume (mm^3^)

The volume of adipose tissue in specific areas was determined by drawing regions of interest (ROIs) on the microCT data using 2D images and interpolated through multiple image slices in order to generate a 3D volume. These measurements were performed by a single operator (to maintain consistency – E.M) using Inveon Research Workplace image analysis software (IRW, ver. 3.0, Siemens Medical Solutions, Knoxville, TN).

### HOMA_IR_

The homeostasis model assessment of insulin resistance (HOMA_IR_) was used as a screening index of changes in insulin sensitivity. HOMA_IR_ is calculated via standard formula from fasting plasma insulin and glucose as follows: HOMA_IR_ = [Insulin (uU/mL) X glucose (mM)]/22.5. The plasma glucose and insulin concentrations were measured using the Glucose Assay Kit from Biovision (Milpitas, CA) and the Insulin kit from Millipore (Billerica, MA), respectively.

### ROS/oxidative stress

Plasma malondialdehyde (MDA) was measured using a fluorometric assay from Zeptrometrix (Buffalo, NY), and plasma 8-isoprostane F_2α_ was measured by using the ELISA kit from Assay Designs (Ann Arbor, MI).

### Inflammatory markers and cytokines

IL-6, adiponectin, MCP-1 and CRP levels in plasma were determined by ELISA (IL-6 and MCP-1: Invitrogen, Grand Island, NY; adiponectin: Millipore: Billerica, MA; CRP: Life Diagnostics, West Chester, PA).

### Statistics

Data were analyzed by one-way ANOVA, and significantly different group means (p < 0.05) were then separated by the least significant difference test. Normality of distribution and homogeneity of variance was confirmed for each data set prior to further analysis.

## Results

**Figure 1:**
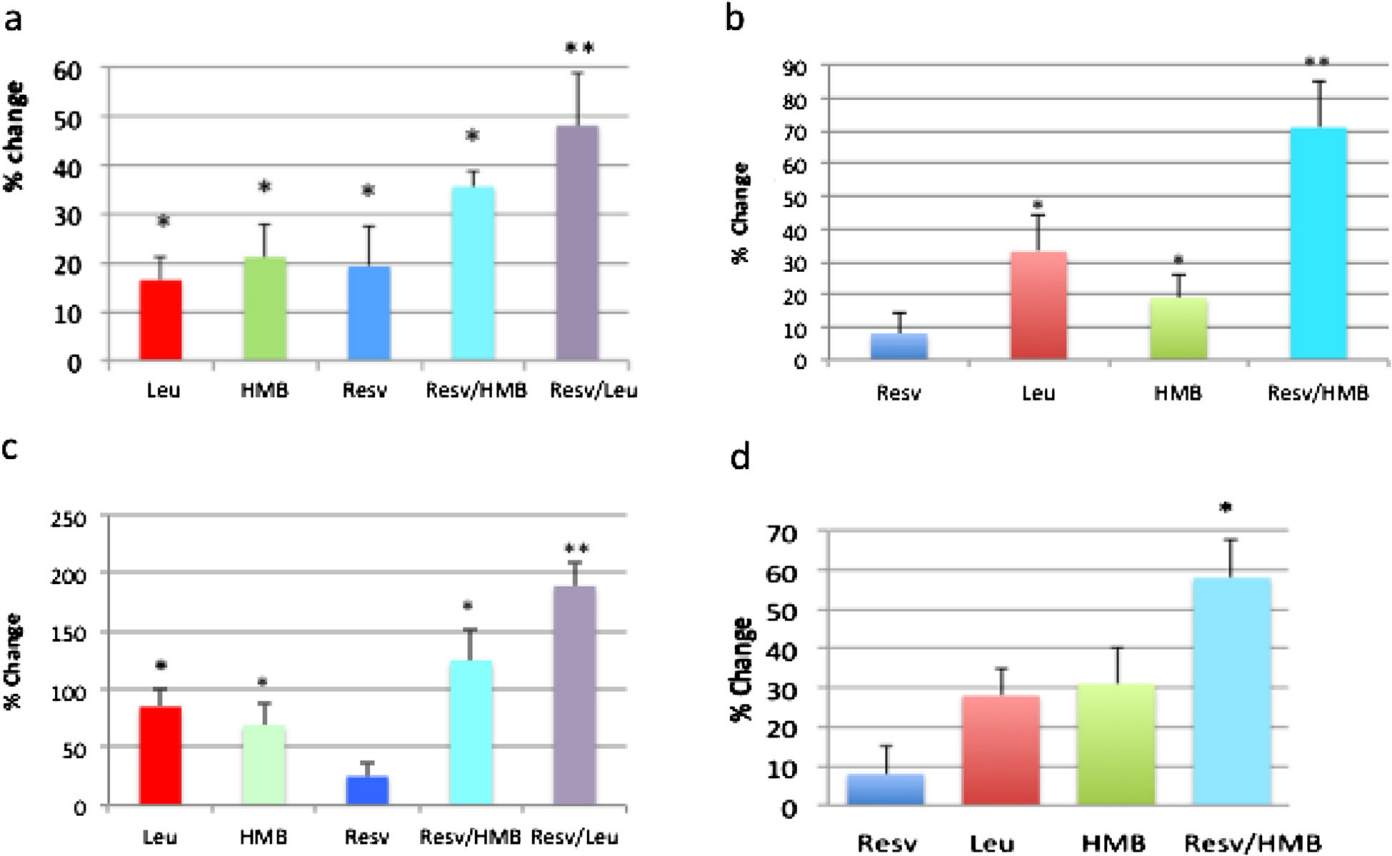
**Leucine and HMB Synergize with Resvetratrol in activation of Sirt1 and Sirt 3 activity. a) Sirt1 in Muscle Cells.** C2C12 muscle cells were incubated with indicated treatments under low glucose (5 mM) conditions and Sirt1 activity was measured. Data are expressed as mean ± SE (n = 7 to 9) and are expressed as % change from control; control = 1085 ± 41 AFU/mg protein. * indicates significant difference compared to control (p < 0.04), ** indicates significant difference compared to control, leucine and HMB (p < 0.01). **b) Sirt1 in Adipocytes.** 3T3-L1 mouse adipocytes were incubated with indicated treatments under low glucose (5 mM) conditions and Sirt1 activity was measured. Data are expressed as mean ± SE (n = 7 to 9) and are expressed as % change from control; control = 759 ± 63 AFU/mg protein. * indicates significant difference compared to control (p < 0.05), ** indicates significant difference compared to control, leucine and HMB (p < 0.01).**c) Sirt3 in Muscle Cells.** C2C12 muscle cells were incubated with indicated treatments under low glucose (5 mM) conditions and Sirt1 activity was measured. Data are expressed as mean ± SE (n = 7 to 9) and are expressed as % change from control; control = 410 ± 57 AFU/mg protein. * indicates significant difference compared to control (p < 0.03), ** indicates significant difference compared to control, leucine and HMB (p < 0.02). **d) Sirt3 in Adipocytes.** 3T3-L1 mouse adipocytes were incubated with indicated treatments for 4 hours under low glucose (5 mM) conditions . Mitochondrial protein was isolated and Sirt3 activity was measured. Data are presented as mean ± SE (n = 6) and are expressed as % change from control; control = 507.8 ± 20.5 AFU/mg protein. * indicates significant difference compared to control (p = 0.03).

Based on our previous data demonstrating the effects of leucine on Sirt1 and mitochondrial biogenesis in muscle cells and adipocytes [5] and the direct effects of leucine and its metabolites HMB and α-ketoisocaproic acid (KIC) on Sirt1 activity in cell-free system [8], we investigated here the synergistic effects of resveratrol with leucine and HMB on Sirt1 and Sirt3 activation in muscle cells and adipocytes (Figure 1). Although resveratrol, leucine and HMB exerted only weak independent effects on Sirt1 (Figure 1 a, b) and Sirt3 (Figure 1 c, d) activity at the indicated concentrations, combining resveratrol with leucine or HMB resulted in an up to ~50% increase in Sirt1 and 3 activity (p < 0.05), with greater effects noted for Sirt3 in muscle cells (~125-175% increases (p < 0.02). 

**Figure 2:**
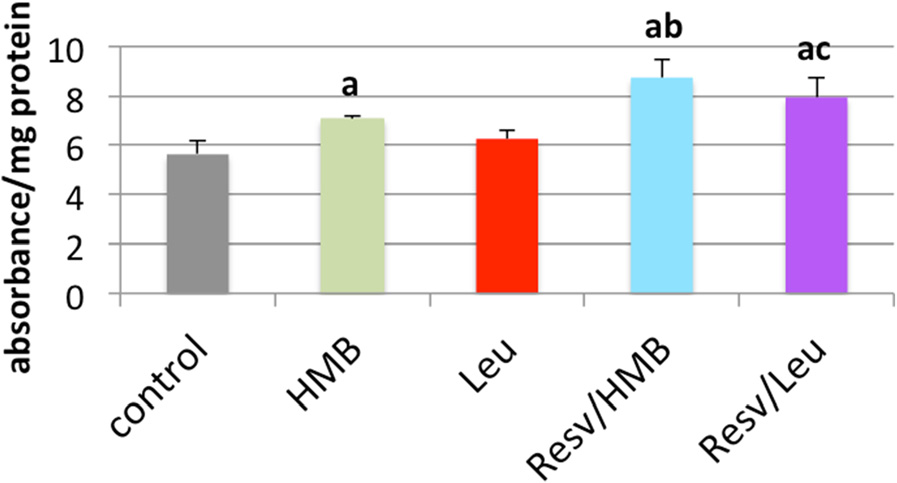
**Leucine and HMB Synergize with Resveratrol to stimulate AMPK activity in 3T3L1 adipocytes.** 3T3-L1 mouse adipocytes were incubated with indicated treatments for 24 hours and AMPK activity was measured. Data are presented as mean ± SE (n = 4). ^a^ indicates significant difference to control (p < 0.04), ^b^ indicates significant difference to control, HMB and leucine (p < 0.03), ^c^ indicates significant difference to control and leucine (p < 0.03).

**Figure 3:**
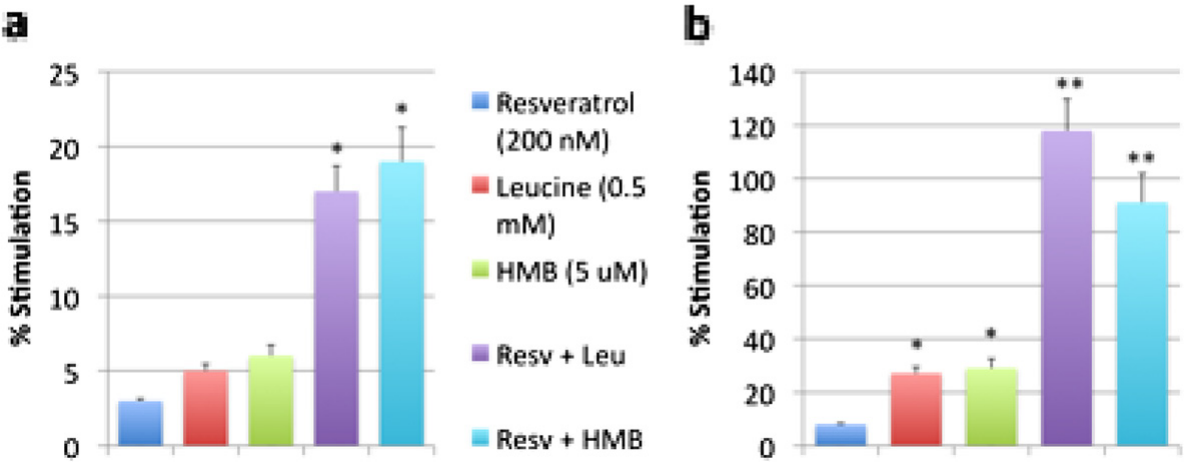
**Leucine and HMB Synergize with Resveratrol to Stimulate Fatty Acid Oxidation under a) Low Glucose Conditions and b) High Glucose Conditions.** 3T3-L1 mouse adipocytes were incubated with indicated treatments for 4 hours under a) low glucose (5 mM) or b) high glucose (25 mM) conditions. Data are presented as mean ± SE (n = 6) and are expressed as % stimulation over control, where low glucose control = 193 ± 39 cpm/ng DNA and high glucose control = 302 ± 24 cpm/ng DNA. Stars above the bars indicate significant difference * compared to control (p < 0.05), ** compared to control, Leucine, and HMB (p < 0.005).

Since there is an interaction between Sirtuins and AMPK, and Sirt1 and Sirt3 exert downstream effects on mitochondrial metabolism and fatty acid oxidation, we also examined the effects of leucine, HMB and resveratrol on AMPK activation and on β-oxidation in adipocytes. AMPK activity was not significantly changed by Leucine and only marginal by HMB alone, while the combination with Resveratrol with either leucine or HMB produced a 42% and 55% increase, respectively (p < 0.03, Figure 2). In the presence of 5 mM glucose, only the combination treatments (200 nM resveratrol plus 5 uM HMB; 200 nM resveratrol plus 0.5 mM leucine) stimulated modest increases in fatty acid oxidation (18%, p < 0.05), while the individual components exerted no independent effect (Figure 3a). Simulating glycemic stress with high (25 mM) glucose medium reduced fatty acid oxidation by 46% compared to low glucose medium (p < 0.05, data not shown); the low dose of resveratrol exerted no effect on fatty acid oxidation under these glucose conditions, but the leucine and HMB exerted modest, but significant effects (27% and 29%, respectively, p < 0.05 vs. control, Figure 3b), and the leucine-resveratrol and HMB-resveratrol combinations each exerted a markedly greater effect (118% and 91% stimulation, respectively; p < 0.005 vs. control and vs. the independent effects of leucine, HMB and resveratrol; Figure 3b). These data demonstrate synergy between resveratrol and leucine or its metabolite, HMB, in stimulation of fat oxidation and promotion of a more oxidative phenotype under conditions that model hyperglycemia.

**Figure 4:**
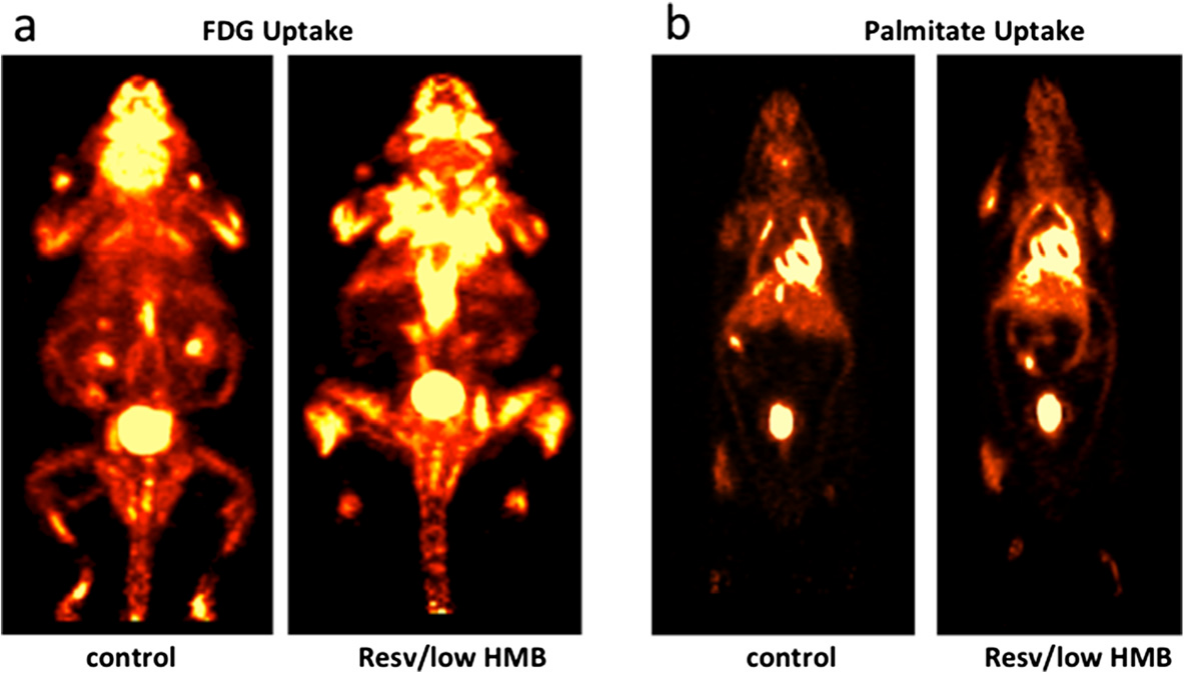
**Resveratrol-HMB synergy in glucose uptake using FDG-PET.** Mice were fed a high fat-diet with indicated treatments for 6 weeks. At the end of the treatment period, Fluorine-18-deoxy-glucose (FDG) or palmitate PET/CT scans were performed to measure whole body glucose (**a**) or fat uptake (**b**). Representative images of control diet group and resveratrol/low HMB diet group are shown

This synergy is consistent with the *in vivo* effects of leucine, HMB and resveratrol on energy metabolism, body composition and insulin sensitivity in diet-induced obese mice. The low doses of resveratrol and HMB exerted no significant independent effect on body weight, weight gain, visceral adipose tissue mass, fat oxidation, respiratory exchange ratio (RER), or heat production, while the high dose of resveratrol significantly increased both heat production and skeletal muscle fat oxidation and decreased RER, indicating a whole-body shift towards fat oxidation (Table 1); however, high dose resveratrol exerted no significant effect on body weight, weight gain, or visceral adipose tissue mass. In contrast to the lack of independent effects of low dose resveratrol or HMB, combining low dose resveratrol with either HMB or leucine resulted in significant reductions in body weight, weight gain, visceral adipose tissue mass, fat oxidation and heat production, and an associated decrease in RER, especially in the dark (feeding) cycle (Table 1). Consistent with this, an increased palmitate uptake in muscle was detected via PET/CT (Table 1; Figure 4b).

**Table 1:** **Effects of resveratrol, leucine and HMB on body weight, weight gain, adiposity and fat oxidation in diet-induced obese mice**^**1**^

	**Control**	**Low Resveratrol**^**1**^	**High Resveratrol**^**2**^	**Low HMB**^**3**^	**Low Resv/ Low HMB**	**Low Resv/ High HMB**^**4**^	**Low Resv/Leucine**^**5**^	**P -value**
**Weight (g)**	40.5 ± 0.5^a^	40.8 ± 2.5^a^	38.7 ± 1.2^a^	40.3 ± 2.1^a^	36.2 ± 3.2^b^	34.4 ± 1.1^b^	38.3 ± 2.3^b^	p < 0.05
**Weight gain (g)**	22.4 ± 1.1^a^	20.9 ± 1.5^a^	22.3 ± 2.4^a^	22.5 ± 1.2^a^	18.2 ± 1.2^b^	19.2 ± 1.0^b^	19.2 ± 1.6^b^	p < 0.01
**Visceral Adipose Volume (mm**^**3**^**)**	6556 ± 143^a^	6551 ± 575^a^	6031 ± 323^a^	6184 ± 460^a^	5302 ± 324^b^	4879 ± 243^b^	4259 ± 321^b^	p < 0.01
**PET palmitate uptake (Muscle SUV)**	1.34 ± 0.15^a^	1.51 ± 0.44^a^	2.29 ± 0.11^b^	1.90 ± 0.29^b^	2.09 ± 0.30^b^	1.97 ± 0.28^b^	1.76 ± 0.09^a,b^	p < 0.05
**Respiratory Exchange Ratio (24 hr RER)**	0.850 ± 0.008^a^	0.847 ± 0.008^a^	0.825 ± 0.007^b^	0.844 ± 0.012^a^	0.815 ± 007^b^	0.818 ± 0.09^b^	0.811 ± 0.010^b^	p < 0.01
**Respiratory Exchange Ratio (Light Cycle)**	0.822 ± 0.013^a^	0.818 ± 0.010^a^	0.803 ± 0.009^b^	0.826 ± 0.011^a^	0.800 ± 0.010^b^	0.811 ± 0.009^ab^	0.799 ± 0.011^b^	p < 0.05
**Respiratory Exchange Ratio (Dark Cycle)**	0.877 ± 0.016^a^	0.876 ± 0.013^a^	0.847 ± 0.011^b^	0.862 ± 0.009^a^	0.830 ± 0.012^b^	0.825 ± 0.014^b^	0.824 ± 0.016^b^	p < 0.02
**Heat Production (kcal/h)**	0.521 ± 0.015^a^	0.517 ± 0.014^a^	0.552 ± 0.015^b^	0.526 ± 0.011^a^	0.544 ± 0.010^b^	0.547 ± 0.009^b^	0.550 ± 0.012^b^	p < 0.05
**Heat Production (kcal/h/kg weight)**	12.86 ± 0.40 ^a^	12.67 ± 0.32 ^a^	14.26 ± 0.38 ^b^	13.05 ± 0.27 ^a^	15.03 ± 0.28 ^b^	15.90 ± 0.23 ^b^	14.36 ± 0.33 ^b^	P < 0.03
**24 h Food intake (day 0 of treatment) (g)**	3.291 ± 0.362	2.931 ± 0.285	3.064 ± 0.406	3.524 ± 0.241	2.810 ± 0.167	3.070 ± 0.319	3.174 ± 0.345	NS
**24 h Food intake (end of 6-weeks) (g)**	3.872 ± 0.346	4.065 ± 0.263	3.598 ± 0.230	3.651 ± 0.331	3.720 ± 0.257	3.794 ± 0.220	3.504 ± 0.280	NS

Mice were fed a high fat-diet with indicated treatments for 6 weeks. Visceral fat volume and muscle palmitate uptake was determined via PET/CT. RER and heat production was calculated from 24 h oxygen consumption measurements in metabolic chambers. Data are presented as means ± SE (n = 5 to 10). Non-matching letter superscripts in each row denote significant differences at the indicated p-value.

^1^Low resveratrol: 12.5 mg resveratrol/kg diet.

^2^High resveratrol: 225 mg resveratrol/kg diet.

^3^Low HMB: 2 g hyroxymethylbutyrate (calcium salt).

^4^High HMB: 10 g hyroxymethylbutyrate (calcium salt).

^5^Leucine: 24 g leucine/kg diet.

None of the dietary treatments exerted any effect on plasma glucose, and neither resveratrol at either dose nor HMB exerted any independent effect on plasma insulin or on muscle glucose uptake (Table 2). However, the combination of low dose resveratrol with either HMB or leucine resulted in significant, marked decreases in plasma insulin. This reduction in insulin with no change in plasma glucose reflects significant improvements in muscle and whole-body insulin sensitivity, as demonstrated by significant and substantial decreases in HOMA_IR_ (homeostatic assessment of insulin resistance) and corresponding increases in skeletal muscle ^18^FDG uptake (Table 2 and Figure 4a). Food intake was measured in the metabolic cages. There was no significant difference in 24 h food intake between groups at either the beginning or the end of the intervention (Table 1).

**Table 2:** Effects of resveratrol, leucine and HMB on indices of insulin sensitivity in diet-induced obese mice

	**Control**	**Low Resveratrol**^**1**^	**High Resveratrol**^**2**^	**Low HMB**^**3**^	**Low Resv/ Low HMB**	**Low Resv/ High HMB**^**4**^	**Low Resv/Leucine**^**5**^	**P value**
**Glucose (mM)**	4.97 ± 0.60	5.14 ± 0.85	5.14 ± 0.75	4.28 ± 0.49	4.67 ± 0.49	4.33 ± 0.41	5.05 ± 0.92	NS
**Insulin (μU/mL)**	12.5 ± 3.4^a^	10.4 ± 1.6^a^	10.1 ± 2.7^a^	8.3 ± 1.1^a^	5.8 ± 0.7^b^	3.9 ± 1.2^b^	5.5 ± 1.4^b^	P < 0.005
**HOMA**_**IR**_	2.61 ± 0.82^a^	2.41 ± 0.66^a^	0.59 ± 0.26^b^	1.93 ± 0.32^a^	1.18 ± 0.25^c^	0.87 ± 0.31^b^	1.14 ± 0.37^c^	P < 0.01
**Muscle Glucose Uptake (**^**18**^**FDG SUV)**	3.64 ± 0.88^a^	3.63 ± 1.29^a^	3.87 ± 0.32^a^	2.99 ± 0.42^a^	5.90 ± 0.41^b^	5.93 ± 1.63^b^	5.68 ± 0.75^b^	P < 0.02

Mice were fed a high fat-diet with indicated treatments for 6 weeks. Muscle glucose uptake was determined via PET/CT. The homeostasis model assessment of insulin resistance (HOMA_IR_) was used as a screening index of changes in insulin sensitivity and was calculated via standard formula from fasting plasma insulin and glucose (HOMA_IR_ = [Insulin (uU/mL) X glucose (mM)]/22.5). Data are presented as means ± SE (n = 5 to 10). Non-matching letter superscripts in each row denote significant differences at the indicated p value.

^1^Low resveratrol: 12.5 mg resveratrol/kg diet.

^2^High resveratrol: 225 mg resveratrol/kg diet.

^3^Low HMB: 2 g hyroxymethylbutyrate (calcium salt).

^4^High HMB: 10 g hyroxymethylbutyrate (calcium salt).

^5^Leucine: 24 g leucine/kg diet.

**Figure 5:**
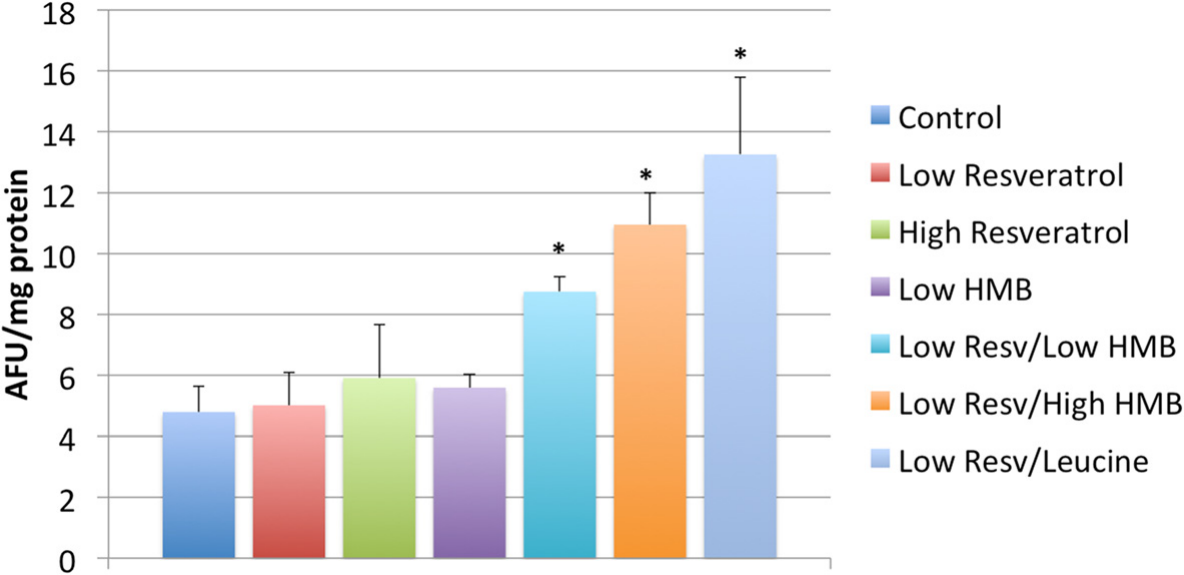
**Effects of resveratrol, leucine and HMB on adipose tissue Sirt1 activity in diet-induced obese mice.** Mice were fed a high fat-diet with indicated treatments for 6 weeks. At the end of the treatment period, Sirt1 activity in adipose tissue was measured. Data are presented as means ± SE (n = 9 to 10). Stars above the bars indicate significant difference compared to control (p < 0.02).

Sirt1 activity in adipose tissue followed a similar pattern (Figure 5). Neither resveratrol nor HMB exerted significant independent effects on Sirt1 activity, although high dose resveratrol exhibited a non-significant trend towards an increase. In contrast, combining low dose resveratrol with either HMB or leucine resulted in ~ two-fold increases in tissue Sirt1 activity; comparable in vivo data are not available for Sirt3 due to limited tissue availability from these mice. Since such sirtuin activation would be anticipated to reduce inflammatory response, we measured inflammatory and oxidative stress markers in the plasma. High dose resveratrol significantly reduced circulating IL-6, while the combination of low dose resveratrol (which exerted no independent effect) with HMB resulted in a markedly greater lowering of IL-6 (Table 3). Similarly, while neither HMB nor low-dose resveratrol exerted any effect on MCP-1 or C-reactive protein (CRP), the combination of low dose resveratrol with either HMB or leucine resulted in significant decreases in both inflammatory biomarkers. Moreover, the anti-inflammatory cytokine adiponectin was increased in response to low-dose resveratrol in combination with either HMB or leucine, while the individual components at these doses exerted no significant effect (Table 3).

**Table 3:** Effects of resveratrol, leucine and HMB on inflammatory biomarkers in diet-induced obese mice

	**Control**	**Low Resveratrol**^**1**^	**High Resveratrol**^**2**^	**Low HMB**^**3**^	**Low Resv/Low HMB**	**Low Resv/High HMB**^**4**^	**Low Resv/Leucine**^**5**^	**P value**
**C-reactive protein (ng/mL)**	95.6 ± 9.6^a^	134.8 ± 8.5^a^	123.9 ± 35.3^a^	98.6 ± 5.1^a^	67.4 ± 12.2^b^	58.3 ± 12.4^b^	55.9 ± 17.7^b^	P < 0.01
**IL-6 (pg/mL)**	29.0 ± 6.4^a^	23.2 ± 2.9^a^	14.1 ± 1.3^b^	19.9 ± 3.1^a^	6.9 ± 1.2^c^	4.5 ± 2.6^c^	11.2 ± 4.1^b^	P < 0.005
**MCP-1 (pg/mL)**	115.8 ± 19.7^a^	104.4 ± 16.5^a^	27.3 ± 6.8^b^	116.8 ± 9.3^a^	24.2 ± 6.2^b^	15.2 ± 3.7^b^	34.9 ± 5.9^b^	P < 0.001
**Adiponectin (ng/mL)**	11.0 ± 0.9^a^	12.4 ± 1.1^a^	14.8 ± 1.8^b^	11.1 ± 1.6^a^	14.1 ± 0.8^b^	16.3 ± 3.0^b^	14.5 ± 1.0^b^	P < 0.03

Mice were fed a high fat-diet with indicated treatments for 6 weeks. At the end of the treatment period, oxidative and inflammatory stress biomarkers in plasma were determined. Data are presented as means ± SE (n = 10). Non-matching letter superscripts in each row denote significant differences at the indicated p value.

^1^Low resveratrol: 12.5 mg resveratrol/kg diet.

^2^High resveratrol: 225 mg resveratrol/kg diet.

^3^Low HMB: 2 g hyroxymethylbutyrate (calcium salt).

^4^High HMB: 10 g hyroxymethylbutyrate (calcium salt).

^5^Leucine: 24 g leucine/kg diet.

## Discussion

In this study we show synergistic actions of either leucine or HMB with resveratrol on Sirt1 and Sirt3 activation and downstream metabolic effects. These effects were found with low concentrations of each compound that can be readily achieved via diet and exert little or no independent effect; however, it should be noted that we relied upon our previous work demonstrating the specifity of leucine over branched chain amino acids [4,5], and did not include other amino acids controls in the present study. Activation of Sirt1 and/or Sirt3 is well recognized to stimulate mitochondrial biogenesis and to activate enzymes involved in glucose and fat metabolism [36,37]. Therefore, Sirt1 activators have been suggested as therapeutic targets for insulin resistance, diabetes and metabolic disease [38,39]. The polyphenol resveratrol has been utilized experimentally as a Sirt1 activator, as it mimics the effects of caloric restriction on lifespan, oxidative and inflammatory stress, insulin sensitivity and adiposity [17,30]. Although its exact mechanism of action is still controversial, resveratrol-induced Sirt1 activation appears to be dose- and time-dependent, and may be dependent upon AMPK activation [19,20]. Park et al. showed that resveratrol inhibits phosphodiesterase 4, resulting in an increase in cAMP and activation of AMPK. This leads to an increase of cellular NAD^+^ levels and subsequent activation of Sirt1 [19]. However, Sirt1-independent AMPK activation may be achieved only at high resveratrol concentration (50 μM) while lower concentrations may activate Sirt1 independently of AMPK activation [20]. Nonetheless, beneficial effects of resveratrol treatment on inflammation, insulin signaling and other metabolic outcomes have been widely reported [40-43]; however, most data come from cell and animals studies while human data are rare and equivocal. It has been suggested that humans exhibit a different bioavailability and metabolism of resveratrol than rodents [25]. Resveratrol is absorbed in limited amounts (about 70%) and rapidly undergoes enterohepatic circulation with extensive sulfation and glucuronidation resulting in up to 20 fold higher plasma levels of conjugated metabolites [27]. Therefore, plasma concentrations of unchanged resveratrol found in humans are usually 10- to 100-fold lower than those used in cell studies (μM range) [27]. Moreover, little is known about the necessary concentrations to achieve physiological effects, bioactivity for resveratrol metabolites, variation of tissue specificity and inter-individual variation of bioavailability [26]. In addition, resveratrol’s effects are dose-dependent and frequently biphasic, with often protective effects at low concentrations (low μM) but adverse effects potentially occurring at higher doses [44]. Similarly, biphasic responses with regard to weight gain were seen in mice fed a high fat diet supplemented with low or high dose of resveratrol [18,36]. While the low dose resveratrol supplementation increased weight gain, the high resveratrol dose decreased the weight gain. However, the opposite results were reported in another study [45]. The low resveratrol concentration used in our cell- and animal studies are about 10- to 100-fold lower than in other comparable studies, and similar to human plasma concentrations (C*max*) achieved after single ingestion of 0.5 g or repeated dose of 150 mg resveratrol [27,29]. We show that the low dose resveratrol is not sufficient to produce any metabolic effects independently while the combination of low dose resveratrol with leucine or HMB exhibited similar effects in vivo on insulin sensitivity, inflammatory markers, fat oxidation and heat production as the high dose resveratrol. In addition, animals fed the combinations, were leaner and gained less weight than the animals fed the high dose resveratrol.

The effects of leucine on adiposity and fat oxidation have been reported previously [4,46,47]. These effects are associated with an increase in mitochondrial biogenesis, mediated, in part, by activation of Sirt1 and Sirt1 dependent pathways [5]. Moreover, leucine and its metabolites appear to activate Sirt1 directly, as shown in a cell-free system, although we cannot completely exclude the possibility that this activation is mediated by the interaction of leucine with the fluorophore [8]. Consistent with our data in this study, leucine supplementation has been demonstrated to attenuate weight gain and inflammatory and oxidative stress and augment insulin sensitivity [48-50].

The interaction between AMPK and Sirt1 is well recognized [51-53]. Therefore, the effects of resveratrol and leucine may be exerted by direct or by indirect activation of Sirt1. Leucine may also stimulate AMPK indirectly by increasing adiponectin levels [48]. Although Sirt1-independent AMPK stimulation by resveratrol has been shown only for high concentrations [19], the low concentration used in this study may be sufficient for AMPK stimulation when combined with small amounts of another AMPK activator such as leucine.

We assessed the diet-treatment effects in live mice by measuring the glucose and palmitate uptake in muscle and adipose tissue using PET/CT imaging. This allowed us to investigate the effects in a whole body system and to visualize the uptake of specific tissues. Although increased substrate uptake does not necessarily equate with increased metabolism and oxidation, these data are in support of our other observations such as increased mitochondrial metabolism, loss of adipose tissue, and increased insulin sensitivity. The uptake of glucose and palmitate was only significantly increased in muscle, and not in adipose tissue. Therefore, it is most likely that the palmitate and glucose were used for further metabolism.

## Conclusion

Collectively, these data demonstrate synergy between low doses of resveratrol and leucine or it’s metabolite HMB in activating Sirt1 and Sirt1-dependent outcomes. These include increased fat oxidation and attenuation of adiposity and obesity, augmentation of insulin sensitivity and reversal of insulin resistance, and attenuation of systemic inflammatory stress. Our data also demonstrate comparable synergy in activating Sirt3 *in vitro*, but insufficient tissue was available to determine whether corresponding synergistic activation of Sirt3 occurred *in vivo*; hence, our *in vivo* conclusions are limited to Sirt1 activation.

## Competing interests

The authors Antje Bruckbauer and Michael B. Zemel are employees and stockholders of NuSirt Sciences, Inc. All others authors declare that they do not have any competing interests.

## Authors' contribution

AB and MBZ designed and directed this study. TT and AB performed all cell and animal experiments except the PET/CT experiments, which were conducted by JW and AS. MA produced the radioisotopes, SK was responsible for isotope injection and animal care during the PET/CT study, EM and DO analyzed the PET/CT data. All authors read and approved the final manuscript.
